# Psychological barriers to adherence to pharmacological treatment of cardiovascular risk conditions in healthcare workers

**DOI:** 10.3389/fpubh.2024.1462281

**Published:** 2024-10-14

**Authors:** Jessica Berenice Flores-Mendoza, Rebeca Robles García, Mirna García-Méndez, Norma Liliana Rodríguez-Argüelles

**Affiliations:** ^1^Facultad de Estudios Superiores Zaragoza, Universidad Nacional Autónoma de México, Mexico City, Mexico; ^2^Centro de Investigación en Salud Mental Global, Instituto Nacional de Psiquiatría Ramón de la Fuente Muñiz, Mexico City, Mexico; ^3^Sindicato Nacional de Trabajadores, Instituto de Seguridad y Servicios Sociales de los Trabajadores del Estado, Mexico City, Mexico

**Keywords:** treatment adherence, stress, depression, attitudes, cardiovascular diseases

## Abstract

**Introduction:**

Cardiovascular diseases (CVD) are the leading cause of death globally. This burden of disease is particularly high among healthcare workers (HCW). However, adherence to treatment of well-known cardiovascular risk conditions (CRC) still represents a challenge, even among healthcare workers (HCW). Since the identification of modifiable related factors is a prerequisite for developing effective public health interventions, the purpose of this study was to develop a predictive model for adherence to pharmacological treatment (APT) for CRC in HCW, using psychological variables related to CVD mortality, such as the type A behavior pattern, perceived stress, depression, anxiety and attitudes toward treatment adherence.

**Methods:**

An anonymous online survey was completed by a non-probabilistic sample of 1,377 Mexican HCW from tertiary public hospitals, with a diagnosis of only one of the following CRC: ischemic heart disease, diabetes, high blood pressure or dyslipidemia. Sociodemographic questionnaires and self-reported measures were used to collect data: PSS-14 for perceived stress, Type A Behavior Pattern Withdrawal Scale, HADS for anxiety and depression symptoms, the Attitudes toward Medication Scale and the Therapeutics Adherence Scale for Patients with Chronic Diseases.

**Results:**

Anxiety and depression symptoms were higher in the group with risk for non-adherence, while perceived stress and positive attitudes toward medication were higher in the group with likelihood of adherence (*p* ≤ 0.05). The Type A behavior pattern and sociodemographic variables did not differ between groups. In a regression model, positive attitudes toward medication and perceived stress doubled APT (OR = 2.04, CI95% = 1.39–2.97; OR = 2.02, CI95% = 1.71–2,39, respectively) whereas depression decreased its likelihood (OR = 0.61, CI95% = 0.58–0.73).

**Discussion:**

In conclusion, psychoeducation for patients with CRC should include information on the advantages of medication for treating their condition, even if they are HCW. Promoting adaptative coping skills to handle daily stressful events, including their CRC, could reduce the level of stress that could increase their APT but also their cardiovascular risk. Moreover, our data provide evidence regarding the importance of identifying and treating depressive symptoms as part of the standard care of this population.

## 1 Introduction

Cardiovascular diseases (CVD), including all diseases of the heart and blood vessels (such as heart attacks and strokes, atherosclerosis, ischemic heart disease, hypertensive diseases, and cardiomyopathy) are the leading cause of death worldwide (1). Ischemic heart disease, diabetes, high blood pressure and dyslipidemia are risk conditions for premature deaths due to CVD (2), which have effective treatment. However, treatment adherence to such cardiovascular risk conditions (CRC) is low in both women and men (3), and among both general population and healthcare workers (HCW), mainly because it involves lifestyle changes, including increasing overall physical activity, reducing alcohol and tobacco use, controlling blood pressure and/or glucose, adopting a heart-healthy diet, and ensuring medication adherence (4–6).

Even HCW are considered to be well informed about etiology, risks and treatment of physical illnesses, they have higher prevalences of such CRC compared to general population (7). Several authors have proposed that this could be explained, at least in part, given the addition of HCW’s occupational exposure to high and chronic stress, which contribute to the development of CVD (8). According to the National Institude for Occupational Safety and Health (9) HCW are exposed to hazards such as working long hours and shifts, and emotional overload due to daily exposure to human suffering and death, and more attention should be pay to elucidate and warn HCW about specific modifiable risk factors that could negatively impact their cardiovascular health.

Negative psychological characteristics related directly or indirectly to CVD mortality might be related also to poor adherence of pharmacological treatment (APT) of CRC such as ischemic heart disease, diabetes, high blood pressure and dyslipidemia among HCW, mainly the so-called *Type A behavior pattern* (TABP) and the individual poor mental health status. TABP is characterized by a strong drive for competitiveness, achievement and recognition, accompanied by tendencies toward hostility, aggression, impatience, extreme urgency, and environmental control (10) involve a coping style that includes excessive use of active strategies and negative emotional responses that trigger and directly increase the mortality of CVD (11–14), due to the constant overload of the sympathetic nervous system (15, 16).

Regarding the poor mental health status, it is well-stablished that depression and anxiety are indirectly associated with CVD mortality (17–21), due to the related lack of adherence to pharmacological and behavioral treatments (22, 23). Unfortunately, these mental health problems are common in those with CVD (24). Indeed, current clinical recommendations emphasize routine mental health screening and treatment in patients with CVD as an integral part of standard care (25–30).

Other candidate factors to consider in order to explain poor adherence to pharmacological treatment in HCW are the lack of knowledge and more importantly the negative attitudes toward health risk conditions and their pharmacological treatment (31). According to several theoretical models of behavioral change, attitudes, as cognitive mediators of behavior change (32–34) should be evaluated and modified if they are negative, to achieve healthy practices and APT (35–41).

Given the above, the objective of the study was to develop a predictive model for APT in a sample of HCW with CRC (ischemic heart disease, diabetes, high blood pressure and dyslipidemia), through perceived stress, type A behavior pattern, depression, anxiety, and attitudes toward APT.

## 2 Method

### 2.1 Participants

This is a descriptive correlational design with a single group of 1,377 Mexican HCW at public tertiary hospitals in Mexico who completed an online survey. The sample was non-probabilistic with standard participants (42) with only one diagnosis (no comorbidity) of the CRC included in the study (ischemic heart disease, diabetes, high blood pressure or dyslipidemia), based on the New York Heart Association (NYHA) criteria (43).

### 2.2 Measures

Sociodemographic variables (gender, marital status, educational attainment), weight (in kg) and health (in cm) were self-reported in an *ad hoc* questionnaire, and body mass index (BMI) was subsequently calculated through the reported weight and height values.

The Type A Behavior Pattern was evaluated using the Spanish version of the Type A Behavior Pattern Withdrawal Scale, validated by Herrera et al. (44) in the Mexican population with CVD, demonstrating a high global Cronbach’s alpha of 0.93, and an explained variance of 67.3%. It is a self-administered tool comprising eight items with five possible answers scored from 0–4. The Type A Behavior Pattern is present if the final score is 24 or more.

Perceived stress was assessed using the Spanish version of the Perceived Stress Scale (PSS-14), validated by Brito-Ortiz et al. (45) in the Mexican population, demonstrating global Cronbach’s alpha of 0.86, and an explained variance of 47%. It is a self-administered questionnaire comprising fourteen items, with five possible answers scored from 0–4. A total score of 14 or less means absence of stress, while 15–28 points implies a perception of occasional stress, 29–42 points means often stressed, and 43 or more points is equivalent to very often stressed.

Anxiety and depressive symptoms were evaluated using the Spanish version of the Hospital Anxiety and Depression Scale (HADS) (46), a self-administered questionnaire comprising 14 items with four possible answers scored from 0–3, for a total of 0–21. In both subscales for anxiety and depression, a score of 0–7 indicated absence of symptoms, 8–10 indicated moderate presence of symptoms, and 11–21 indicated significant presence of symptoms. This version for Mexican patients with a CVD yielded a global Cronbach’s alpha of 0.90 (anxiety subscale *α* = 0.87, and depressive subscale *α* = 0.77), and an explained variance of 46.94%.

Attitudes toward pharmacological treatment were evaluated using the Attitudes toward Medication Scale (47), a scale designed in Spanish especially for Mexican patients that has an adequate overall reliability index (*ω* =0.907) and an explained variance of 67.5%. It comprises 21 evaluative bipolar adjectives, with seven response options scored from 1–7. Based on the cut-off points of the instrument, attitudes are considered negative if the score is 15–64, while attitudes toward adherence to pharmacological treatment are considered positive if the score is 65–147.

Finally, APT was evaluated using the Spanish version of the Therapeutic Adherence Scale for Patients with Chronic Diseases, validated by Soria, Vega and Nava (48), which has a global Cronbach’s alpha of 0.91 and an explained variance of 41.1%. This is a self-administered tool comprising 24 items, with three response options ranging from 1–3. Based on the cut-off points established in the instrument by the authors, a greater risk of non-adherence is considered if scores are between 24 and 48, while a greater possibility of adherence is reflected with scores between 49 and 72.

### 2.3 Procedure and data analyses

The instruments were administered through a national online cardiovascular mental health session, in which a total of 2,051 HCW were invited to participate in the study. Four hundred and eighty-nine were excluded for failing to meet the inclusion criteria, and 185 removed for having more than one CVC. Evaluation was anonymous, through Google Forms and lasted 30 min. At the beginning of the assessment, those who agreed to participate were asked to electronically sign the informed consent form and given instructions for each instrument and their respective response options. The survey remained active for a month.

Descriptive statistics were used to characterize the study sample, including mean, standard deviation, and range for continuous variables, and frequencies and percentages for categorical variables. Shapiro–Wilk test was used to confirm that data set was well-modeled by a normal distribution, as it has proven to be a powerful normality test for large samples size (49). Therefore, comparisons of age and treatment adherence by CRC were performed using one-way analyses of variance (ANOVA) with Bonferroni correction (for), as the typical method for comparing three or more groups means in clinical studies (50). Comparisons of categorical variables by CRC were warried out using Pearson’s chi-square tests, which might be used to examine this type of variables when the sample size is large (51).

Psychological variables were compared by adherence groups (1 = with higher risk of non-adherence, and 2 = with higher likelihood of adherence) using independent samples Student’s t test. Those showing a significant difference between those with higher risk of non-adherence, and higher likelihood of adherence were included as possible predictors in a logistic regression analysis, using APT as the dependent variable. The Hosmer-Lemeshow goodness-of-fit test was conducted to prove the final model. In all cases, data with *p* < 0.05 were considered statistically significant.

With our sample size, according to G*power calculation, a power of 95% was obtained (alpha = 0.05, two tails).

## 3 Results

The final study sample comprised 1,377 HCW with a diagnosis of either ischemic heart disease (*n* = 554, 40.23%), diabetes (*n* = 341, 24.76%), hypertension (*n* = 242, 17.57%) or dyslipidemia (*n* = 240, 17.44%). Table 1 provides a description of the sociodemographic variables, BMI and treatment adherence in the total sample and by CRC.

**Table 1:** Sociodemographic variables, BMI and pharmacological treatment adherence by CRC.

Variable	Total Sample (*n* = 1,377)	Ischemic Heart Disease (*n* = 554)	Diabetes (*n* = 341)	Hypertension (*n* = 242)	Dyslipidemia (*n* = 240)	Comparisons
Age, mean ± SD	43.41 ± 10.84	44.32 ± 12.39	47.78 ± 10.34	40.87 ± 9.62	42.32 ± 9.12	(*F = 20.11, gl = 4, p = 0.000*)
Range	18–87	18–87	22–82	18–66	19–65	
Gender, *n* (%)						
Women	757 (55%)	288 (52%)	181 (53%)	189 (78%)	122 (51%)	(*X^2^ = 0.065, gl = 4, p = 0.851*)
Men	620 (46%)	266 (48%)	160 (47%)	53 (22%)	118 (49%)	
Marital status						
Single	560 (40.7%)	204 (36.8%)	123 (36.1%)	103 (42.6%)	108 (45%)	(*X^2^ = 3.86, gl = 4, p = 0.695*)
Partnered	817 (59.3%)	350 (63.2%)	218 (63.9%)	139 (57.4%)	132 (55%)	
Education						
Undergraduate	250 (18.1%)	93 (16.8%)	60 (17.5%)	51 (21%)	48 (20%)	(*X^2^ = 1.38, gl = 4, p = 0.986*)
Bachelor	715 (51.9%)	288 (52%)	211 (62%)	148 (61.2%)	140 (58.2%)	
Master or more	412 (30%)	173 (31.2%)	70 (20.5%)	43 (17.8%)	52 (21.8%)	
BMI						
Normal weight	295 (21.42%)	115 (20.8%)	60 (17.6%)	83 (34.3%)	65 (27.1%)	(*X^2^ = 5.15, gl = 4, p = 0.524*)
Overweight	216 (15.7%)	80 (14.4%)	52 (15.2%)	57 (23.6%)	42 (17.5%)	
Obesity	866 (62.88%)	359 (64.8%)	229 (67.3%)	102 (42.1%)	133 (55.4%)	
Treatment adherence, mean ± SD	54.15 ± 7.14	55.11 ± 6.12	54.13 ± 7.02	55.11 ± 6.14	53.17 ± 6.95	(*F = 2.33, gl = 4, p = 0.801*)
Range	24–72	24–72	24–72	24–72	24–72	
Higher risk of non-adherence	216 (15.7%)	86 (15.5%)	45 (13.2%)	38 (15.5%)	43 (17.9%)	
Higher likelihood of adherence	1,161 (84.3%)	468 (84.5%)	295 (86.8%)	204 (84.5%)	197 (82.1%)	

In general, approximately half of the participants was women (*n* = 757, 55%) and the mean age of the total sample was 43.41 ± 10.84 (range = 18–87) years old. Around 60 % had a partner, and the majority has completed the bachelor’s degree or more (master or doctorate). Only around 20 % of the participants has a Body Mass Index (BMI) indicative of normal weight. Concerning APT, 15.7% (*n* = 216) had higher risk of non-adherence, and 1,161 (84.3%) reported higher likelihood of adherence.

As can be seen, there were not significant differences in most of sociodemographic variables and the total score of treatment adherence (our dependent variable). Thus, further analyses were using all participant as a single group.

Regarding psychological variables, it highlights that 15% (*n* = 207) of the sample reported Type A personality, more than 70 % has moderate to severe anxiety symptoms (moderate: *n* = 244, 17.7% and severe: *n* = 735, 53.4%), around 40 % reported moderate to severe depressive symptoms (moderate: *n* = 366, 26.6%; severe: *n* = 164; 11.9%), and more than 60% perceived to be frequent or very frequent stressed (frequently: *n* = 766; 55.6%, very frequently: *n* = 102; 7.4%).

The mean score in attitudes toward treatment adherence was 91.5 ± 17.7 (range = 16–134); around 10 % (*n* = 142; 10.3%) with a score indicative of negative attitudes toward treatment adherence, and *n* = 1,235 (89.7%) with positive attitudes toward treatment adherence.

Table 2 offers descriptives and the results of the comparisons of these psychological variables by groups of APT: (1) with higher risk of non-adherence, and (2) with higher likelihood of adherence. As can be seen, anxiety and depression were higher in the group with risk for non-adherence, while perceived stress and positive attitudes toward medication were higher in the group with likelihood of adherence.

**Table 2:** Psychological variables and attitudes toward medication by groups of treatment adherence.

Variable	Higher risk of non-adherence (*n* = 216)		Higher likelihood of adherence (*n* = 1,161)			
	*M*	*SD*	*M*	*SD*	*t*	*p*
The type A behavior pattern	18.68	4.06	19.25	4.27	−2.16	0.030
Anxiety	2.37	0.81	2.22	0.88	2.76	0.006
Depression	1.74	0.79	1.44	0.66	7.0	0.000
Perceived Stress	25.98	11.80	31.40	9.35	−8.86	0.000
Attitudes toward medication	86.43	19.91	92.45	17.22	0.5.44	0.000

Only the Type A behavior pattern did not differ between groups; therefore, it was excluded for further regression analysis. The same happened with sociodemographic variables (age, gender, marital status, education) and BMI, which did not differ statistically between the non-adherence risk group and the group with a higher likelihood of adherence.

Table 3 shows the results of the logistic regression analysis. As can be seen, positive attitudes toward medication and perceived stress doubled APT (OR = 2.04, CI95% = 1.39–2.97; OR = 2.02, CI95% = 1.71–2.39, respectively) whereas depression decreased its likelihood (OR = 0.61, CI95% = 0.58–0.73).

**Table 3:** Psychological predictors of pharmacological treatment adherence (*n* = 1,377).

	Estimate	Standard error	*Z*-Value	Pr(>|z|)	Odds Ratio	2.50%	97.50%
(Intercept)	−0.546	0.539	−1.012	0.312	0.579	0.202	1.674
Depression	−0.494	0.094	−5.269	≤0.0001**	0.610	0.508	0.733
Perceived stress	0.704	0.086	8.200	≤0.0001**	2.023	1.710	2.396
Positive attitudes toward medication	0.716	0.194	3.693	≤0.0001**	2.046	1.390	2.975

*n* = 1,377; **p ≤ 0.01; R^2^ Nagelkerke = 0.116; Hosmer-Lemeshow goodness-of-fit test: 3.7719, *p* > 0.05.

## 4 Discussion

Although effective pharmacological treatment for CRC such as ischemic heart disease, diabetes, high blood pressure and dyslipidemia is available, APT is a challenge for many patients. Given that the identification of modifiable related factors is a prerequisite for developing effective interventions to increase complex healthy behaviors such as APT, and the need for interventions including psychosocial aspects to prevent HCW’s CVD risks more effectively (52), this study sought to determine the psychological variables that predict the APT of these CRC in a sample of Mexican HCW, an specific population with increased cardiovascular risk (53).

Potential psychological predictors of APT among HCW include those systematically associated with CVD mortality according to the literature in the field: Type A behavior pattern, perceived stress, depression, anxiety, and attitudes toward medication (10, 11, 13–16, 54, 55).

According to our data, positive attitudes toward medication and perceived stress double the probability of APT, while depressive symptoms decrease its probability. In line with previous studies (32–34, 40, 46, 56), attitudes toward healthy practices and treatment play a key role in achieving and maintaining the APT of CRC in Mexican HCW. In this study, positive attitudes toward medication were found to increase APT, highlighting the importance of efforts to boost them rather than merely identifying and modifying negative attitudes (such as fear of negative side effects). Interventions to improve APT for HCW with CRC should therefore strategies to promote positive attitudes toward medication, including the analysis of the advantages of medication for treating their condition.

Interestingly, perceived stress, which is a relevant occupational issue among HCW (8, 9), increased APT for CRC in our sample. This might reflect that stress could prompt active coping with daily stressful events in this specific population, including their CRC and treatment. However, it has been clearly established that chronic high stress levels increase the probabilities of CVD mortality (26–28). Promoting more adaptative coping skills for dealing with these daily stressful events could replace the need for a stressful approach to solving problems, (including CRC though APT) while HCW could also learn evidence-based techniques for stress management (57).

The findings regarding the negative impact of depressive symptomatology on APT for CRC in our sample are in line with prior evidence on depression as a variable that increases CVD mortality due to a related lack of adherence to pharmacological and behavioral treatments (18, 22, 23, 29), and contributes evidence on the importance of identifying and treating depressive symptoms as part of the standard care of patients with CRC (19, 30), mainly in HCW, which compared with other groups of population, have a higher prevalence of depression (58).

Finally, we realize that although a large sample of HCW with CRC was recruited, our study has limitations, including that all information is based solely on self-reports. Moreover, its cross-sectional design makes it impossible to generalize or determine causal links between the variables studied. Additionally, the type of HCW (e.g., nurses, physicians, etc.) was not considered in the present study as variables that can affect directly or indirectly the APT of CRC. Future studies with more objective evaluations, a longitudinal design exploring the effectiveness of the psychological techniques proposed to modify the impact of attitudes toward medication, perceived stress and depressive symptoms on the APT of CRC, as well as comparing the results by type of HCW should be conducted to corroborate and expand our findings.

In conclusion, according to this study among Mexican HCW from tertiary public hospitals with a diagnosis of one CRC (ischemic heart disease, diabetes, high blood pressure or dyslipidemia), some psychological variables related to CVD mortality also impact on APT of CRC, mainly positive attitudes toward medication and perceived stress, which doubled APT for CRC, and depression, which decreased its likelihood.

## Data Availability

The original contributions presented in the study are publicly available. This data can be found here: https://figshare.com/s/fa462efb9684ccca3737.
